# Supplementary material for: Tick extracellular vesicles enable arthropod feeding and promote distinct outcomes of bacterial infection
Source: Nat Commun. 2021 Jun 17;12:3696. doi: 10.1038/s41467-021-23900-8 (PMC8211691; doi:10.1038/s41467-021-23900-8)
Supplement: Supplementary file 1 — Supplementary Information [file 41467_2021_23900_MOESM1_ESM.pdf]

Supplementary Information

# Tick Extracellular Vesicles Enable Arthropod Feeding and Promote Distinct Outcomes of Bacterial Infection

Adela S. Oliva Chávez, Xiaowei Wang, Liron Marnin, Nathan Archer, Holly L. Hammond, Erin E. McClure Carroll, Dana K. Shaw, Brenden G. Tully, Amanda D. Buskirk, Shelby L. Ford, L. Rainer Butler, Preeti Shahi, Kateryna Morozova, Cristina C. Clement, Lauren Lawres, Anya J. O'Neal, Choukri Ben Mamoun, Kathleen L. Mason, Brandi E. Hobbs, Glen A. Scoles, Eileen M. Barry, Daniel E. Sonenshine, Utpal Pal, Jesus G. Valenzuela, Marcelo B. Sztein, Marcela F. Pasetti, Michael L. Levin, Michail Kotsyfakis, Steven M. Jay, Jason F. Huntley, Lloyd S. Miller, Laura Santambrogio, Joao H.F. Pedra

Correspondence to: jpedra@som.umaryland.edu

**This PDF file includes:**

Supplementary Figures 1-12

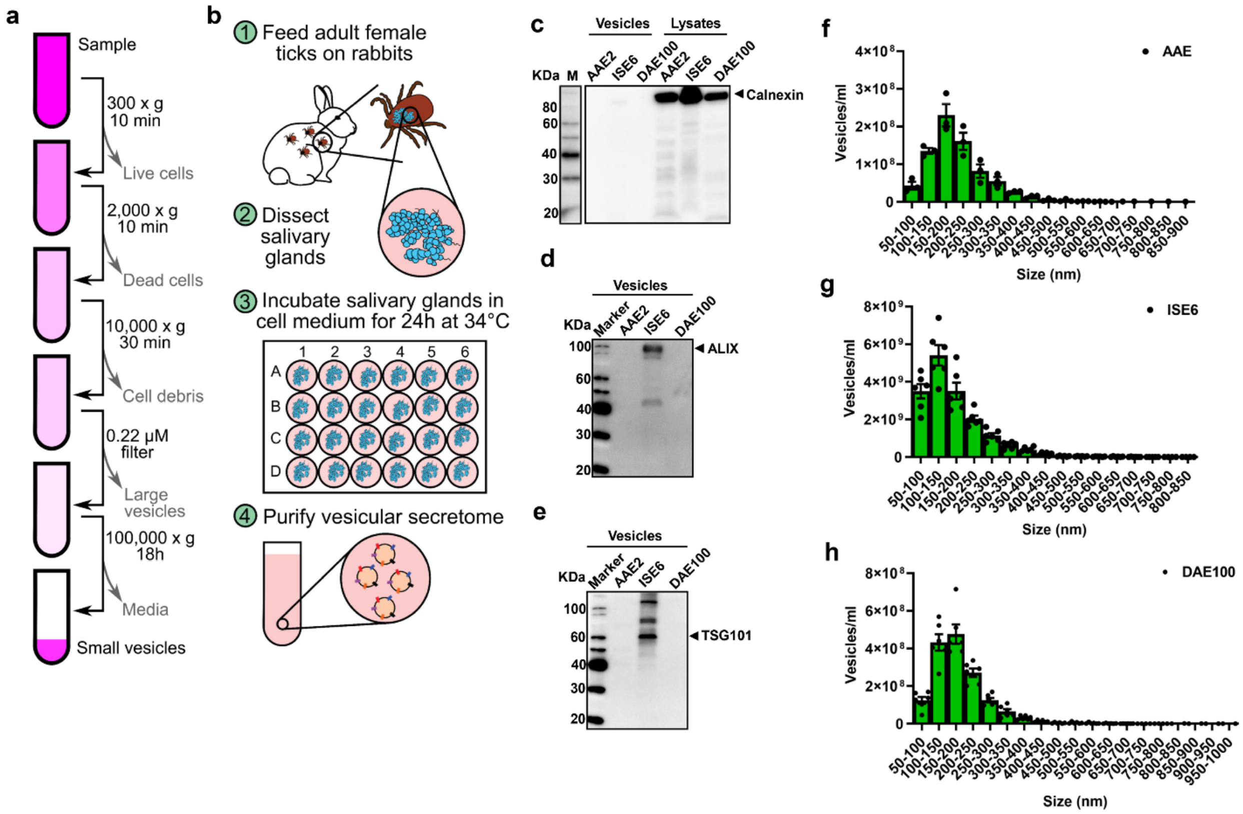


**Supplementary Figure 1.** Tick cell lines secrete EVs. **a** EV purification methodology in ticks. **b** Salivary gland EV production. SDS-PAGE immunoblots show **c** Calnexin$^-$, **d** ALIX$^+$, and **e** TSG101$^+$ EVs purified from uninfected AAE2, ISE6, and DAE100 cells (70 µg of protein). The image is representative of two independent experiments. EVs from **f** *A. americanum* (AAE2) cells, **g** *I. scapularis* (ISE6) cells, and **h** *D. andersoni* (DAE100) cells. Release was measured by nanoparticle tracking analysis. Mean ± standard error of the mean (SEM) is plotted. Data represent three or six technical replicates from one of two independent experiments. Source data are provided as a Source Data file.

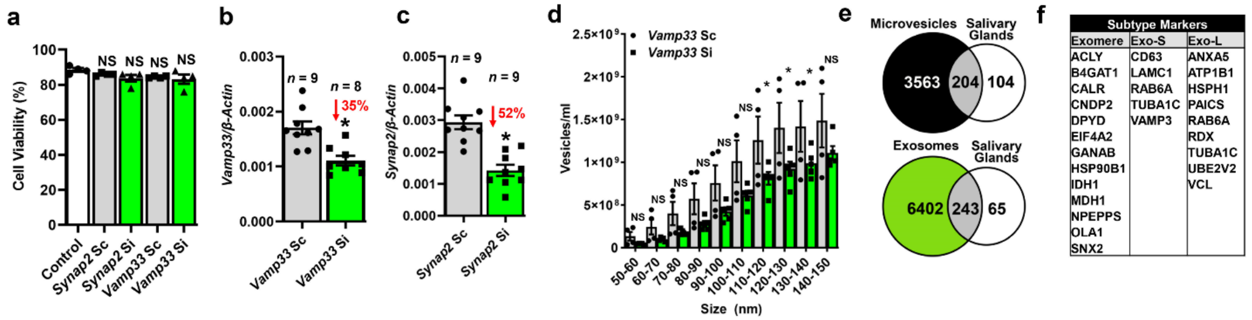


**Supplementary Figure 2.** SNAREs regulate the biogenesis of tick EVs. **a** ISE6 (1x$10^6$) cells were treated with *Synaptobrevin 2* (*Synap2*) and *Vamp33* silencing (Si; green) or scramble (Sc; gray) RNA. Cell viability was measured with Trypan Blue and compared to non-transfected cells (control; white). Mean ± standard error of the mean (SEM) is plotted. Differences in viability were analyzed with One-way ANOVA (NS= not significant). The graph represents the average from two independent experiments combined. **b** and **c** RNA silencing levels in ISE6 tick cell lines. Mean ± SEM are plotted. Statistical differences were evaluated by Two-tailed Student's *t* test (*$p$<0.05); *n*= number of wells analyzed. **d** ISE6 cells (2x$10^7$ cells) were transfected with small interfering RNA against *Vamp33* (Si; green) or scramble control (*Vamp33* Sc; gray) and EVs were measured by nanoparticle tracking analysis. Mean ± SEM from technical replicates of one of two independent experiments are plotted. Both independent experiments show similar results. Two-way ANOVA using size and treatment as variables followed by Fisher's least significant difference statistical test comparing vesicles of the same size between treatments. *$p$<0.05. NS = not significant. **e** and **f** microvesicle and exosomal subtype markers in EVs derived from *I. scapularis* salivary glands (Supplementary Data 2). Source data are provided as a Source Data file.

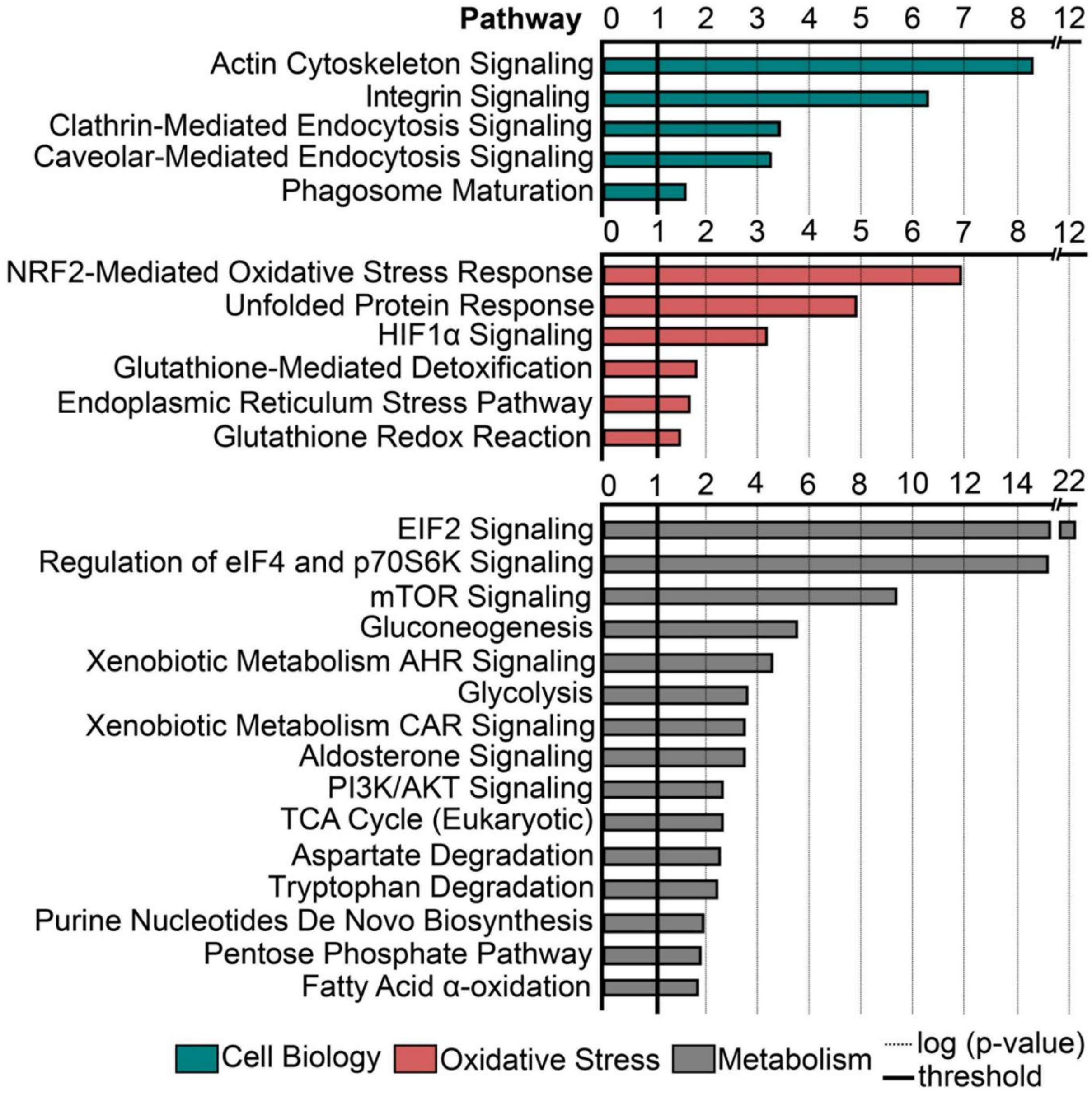


**Supplementary Figure 3.** Biochemical networks enriched in EVs derived from *I. scapularis* salivary glands. Proteins identified within EVs originated from *I. scapularis* salivary glands were used to determine overrepresentation based on the ingenuity pathway analysis. Supplementary Data 2 is also provided. The biological relationship between proteins was determined using right-tailed Fisher's exact test with Benjamini-Hochberg multiple-testing correction. $p<0.05$; −log ($p$ value) > 1.5.

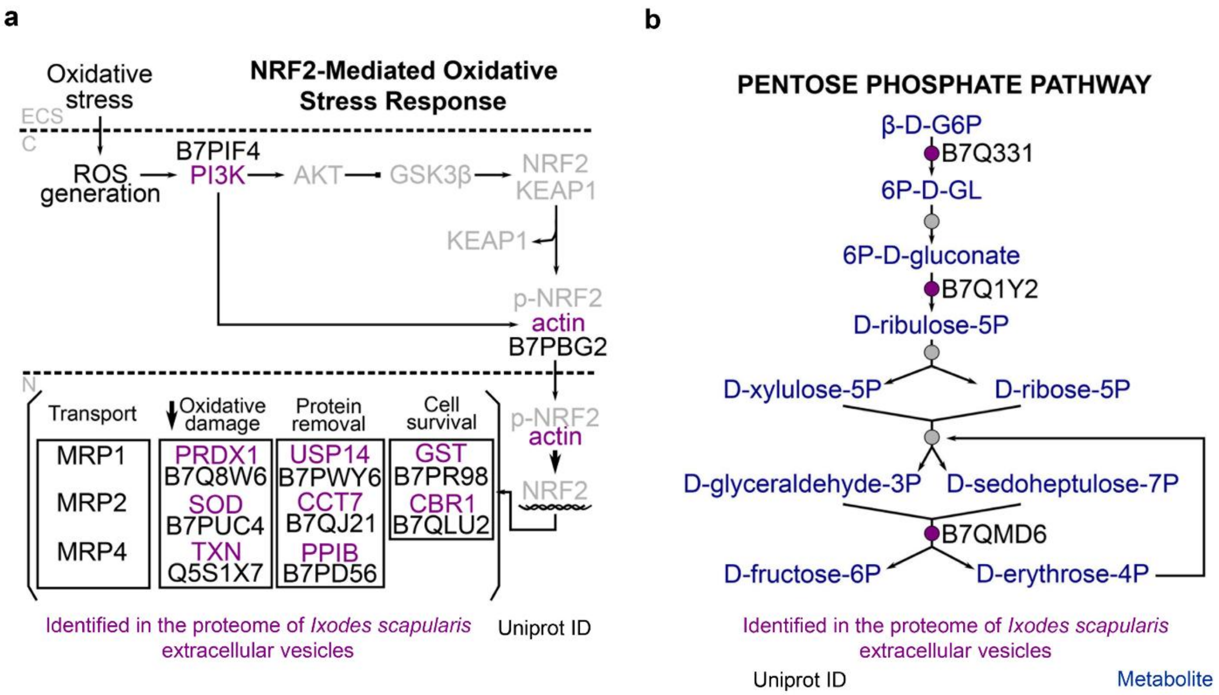


**Supplementary Figure 4.** Overrepresentation of the nuclear factor E2-related factor 2 (NRF2) and the pentose phosphate pathways in the *I. scapularis* EV proteome. **a** NRF2 signaling pathway: ECS, extracellular space; C, cytosol; N, nucleus; ROS, reactive oxygen species; PI3K, phosphoinosotide kinase 3; AKT, protein kinase B (also known as PKB); GSK3β, glycogen synthase kinase 3β; KEAP1, kelch-like-EHC-associated protein 1; p, phosphorylated; GST, glutathione-S transferase; CBR1, carbonyl reductase 1; USP14; ubiquitin carboxyl-terminal hydrolase 14; CCT7, chaperonin-containing T-complex protein 1 (TCP1) subunit 7; PPIB, peptidyl-prolyl cis-trans isomerase B; PRDX1, Peroxiredoxin 1; SOD, superoxide dismutase; TXN, Thioredoxin; MRP1, multidrug resistance-associated protein 1; MRP2, multidrug resistance-associated protein 2; MRP4, multidrug resistance-associated protein 4. **b** Pentose phosphate pathway: β-D-G6P, β-D-glucose-5-phosphate; 6P-D-GL, 6-phosphate-D-gluconolactone; 6P-D-gluconate, 6-phospho-D-gluconate; D-ribulose-5P, D-ribulose-5-phosphate; D-xylulose-5P, D-xylulose-5-phosphate; D-ribose-5P, D-ribose-5-phosphate; D-glyceraldehyde-3P, D-glyceraldehyde-3-phosphate; D-sedoheptulose-7P, D-sedoheptulose-7-

phosphate; D-fructose-6P, D-fructose-6-phosphate; D-erythrose-4P, D-erythrose-4-phosphate. Arrowhead, acts on, stimulatory; square arrowhead, acts on, inhibitory; circular arrowhead, catalyzes; line arrowhead, inhibits. Purple circles and letters indicate proteins that were found in the proteome of *I. scapularis* salivary gland EVs. Gray circles and letters are proteins missing from the pathway. Supplementary Data 5 is also provided.

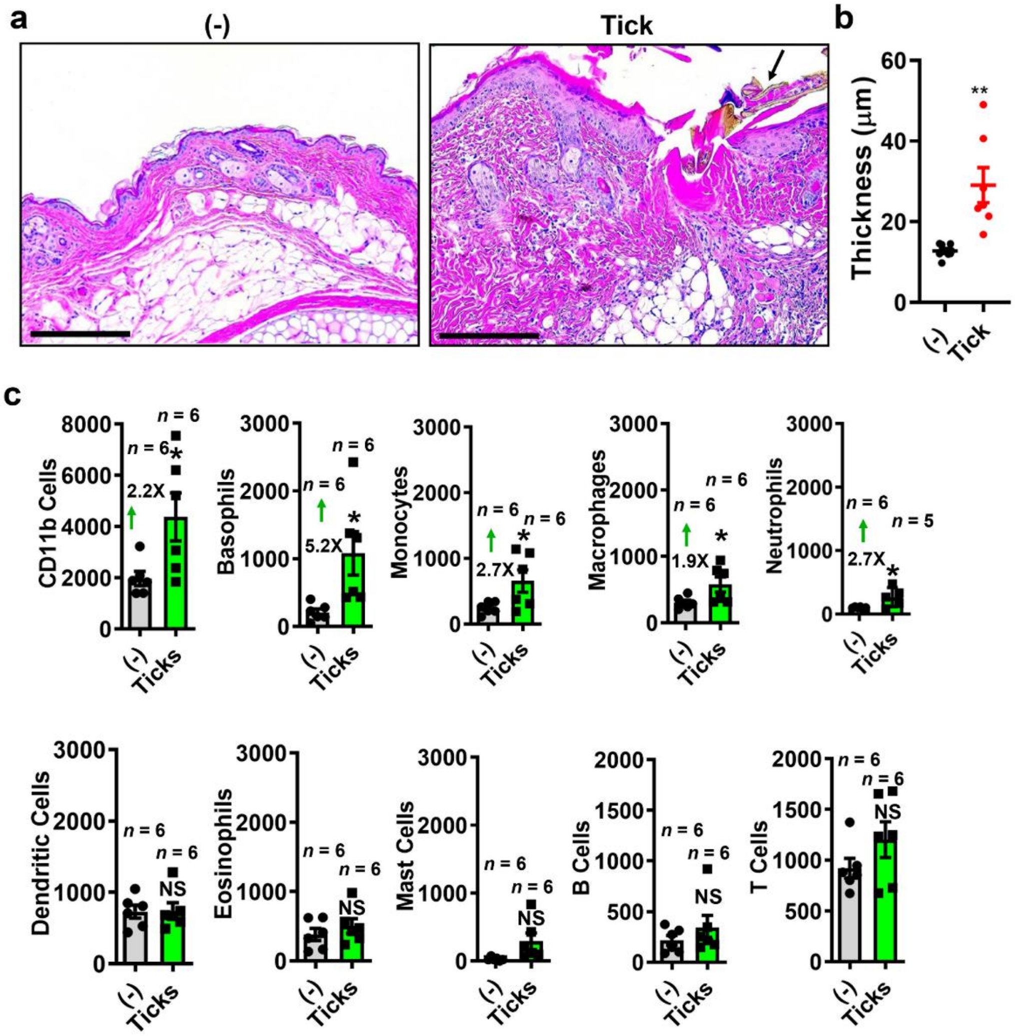


**Supplementary Figure 5.** Tick feeding causes inflammation at the bite site. Ticks were placed on wildtype C57BL/6 mice and allowed to feed for 3 days. Control skin biopsies (-) were taken from non-infested mice at similar locations. **a** Hematoxylin and eosin (H&E) staining displays baseline conditions at the skin site, as depicted by low cell infiltration and a single cell layer in the epithelium. Conversely, tick feeding increased cell infiltration to the bite site and resulted in epithelial damage and thickening of the skin. Arrow indicates the tick hypostome inserted at the bite site (bar = 200 μm). **b** Changes in epidermal thickness due to tick feeding. Skin biopsies from tick-infested mice (red) displayed an increase in thickness when compared to control samples (black); data points represent the thickness across different measures per sample. *n* = number of skin samples analyzed. Data is presented as a mean and standard error of the mean

(± SEM). Statistical differences were evaluated with a two-tailed student's *t* test. (*$p<0.05$) **c** Ticks (green) fed on mice for three days. Skin biopsies from control and tick-infested treatments revealed increased inflammation and infiltration of myeloid cells at the skin site, as determined by flow cytometry. The H&E staining of the skin is a representative picture derived from 8 biological replicates. Data is presented as a mean and standard error of the mean (± SEM). Statistical differences in immune cell populations in the flow cytometry experiments were evaluated by the two-tailed Student's *t* test (basophils $p$=0.023; CD11b $p$= 0.03; monocytes $p$= 0.039; neutrophils $p$= 0.025; and macrophages $p$=0.045; NS = not significant); $n$ = number of skin samples analyzed. Source data are provided as a Source Data file. Gating strategy in flow cytometry experiments is shown in Supplementary Fig. **12**.

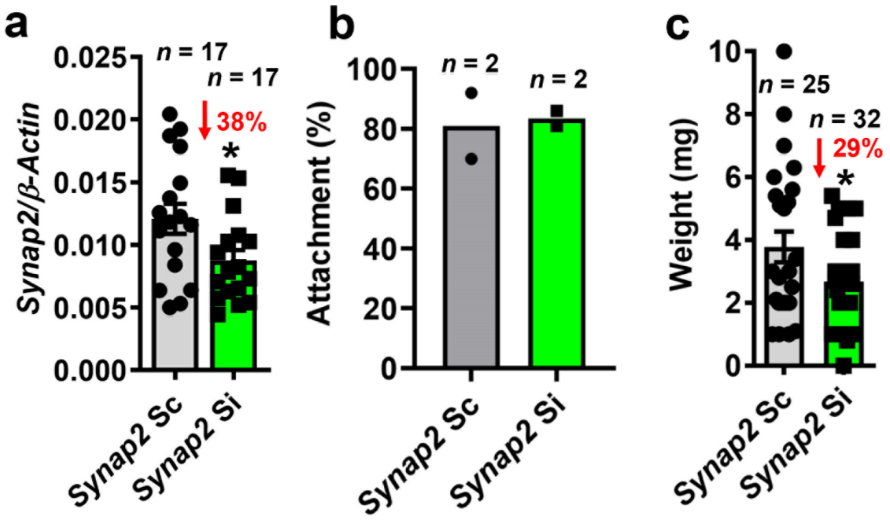


**Supplementary Figure 6.** *Ixodes scapularis synaptobrevin 2* affects feeding at the bite site. **a** Ticks were microinjected with *synaptobrevin 2* small interfering RNA (*synap2 si*) (green) or scrambled RNA (*synap2 sc*) (gray) to reduce the EV release. Gene silencing was determined by quantitative real time PCR in non-engorged ticks 24 hours post-microinjection. In **b** and **c** the attachment and weight of ticks were measured after three-day placement on C57BL/6 mice. Attachment and weight of *I. scapularis* ticks microinjected with *synaptobrevin 2* small interfering (*Synap2 si*) (green) (*n*=32) or scrambled (*Synap2 sc*) (gray) (*n*=25) RNA on C57BL/6 mice (*n*=2 each treatment). Graph indicates results from two independent experiments. Data is presented as a mean and standard error of the mean (± SEM). Statistical differences in gene silencing and attachment were evaluated by the Student's two-tailed *t* test, whereas tick weight was assessed by the one-tailed *t* test. (Silencing (**a**) *p*=0.029; weight (**c**) *p*=0.035) ; NS = no significant); *n* = number of ticks or mice analyzed. Source data are provided as a Source Data file.

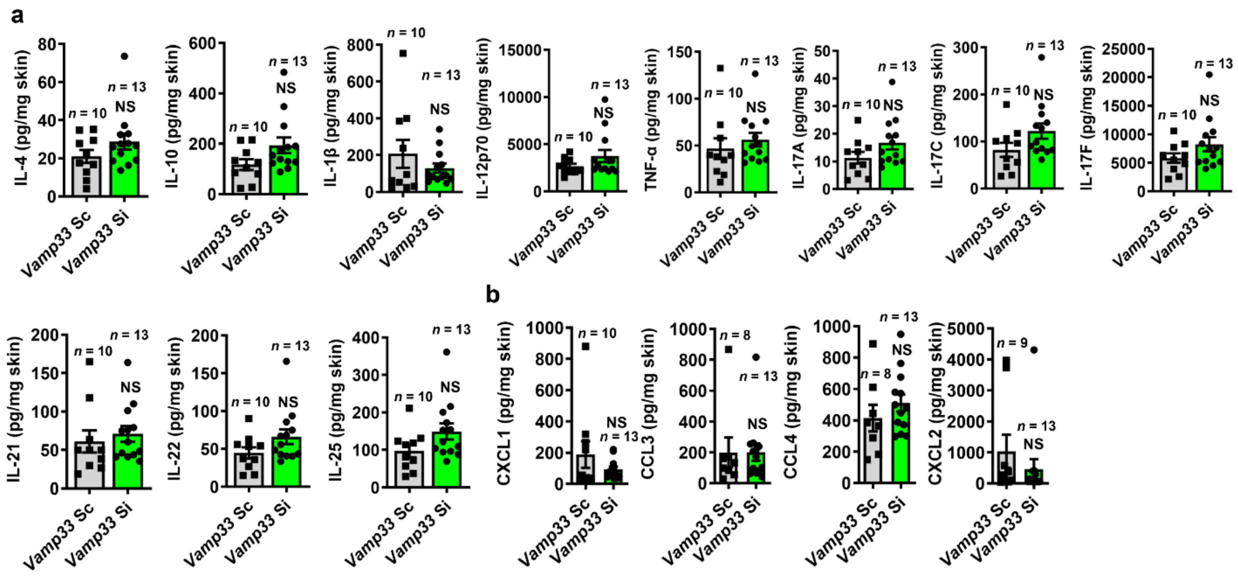


**Supplementary Figure 7.** Cytokines and chemokines at the *I. scapularis* bite site. Ticks were microinjected with *vamp33* small interfering RNA (*vamp33 si*) (green) or scrambled RNA (*vamp33 sc*) (gray) to reduce the EV release. Ticks were placed on C57BL/6 mice and allowed to feed for three days. In **a** and **b**, skin biopsies were taken from the area of feeding, weighted, and homogenized for cytokine and chemokine measurements, respectively. Data are presented as a mean and standard error of the mean (± SEM). Statistical differences in treatments for the cytokine and chemokine measurements at the skin site were evaluated by the Student's two-tailed *t* test. (*$p$<0.05; NS = not significant); $n$ = number of samples analyzed. Source data are provided as a Source Data file.

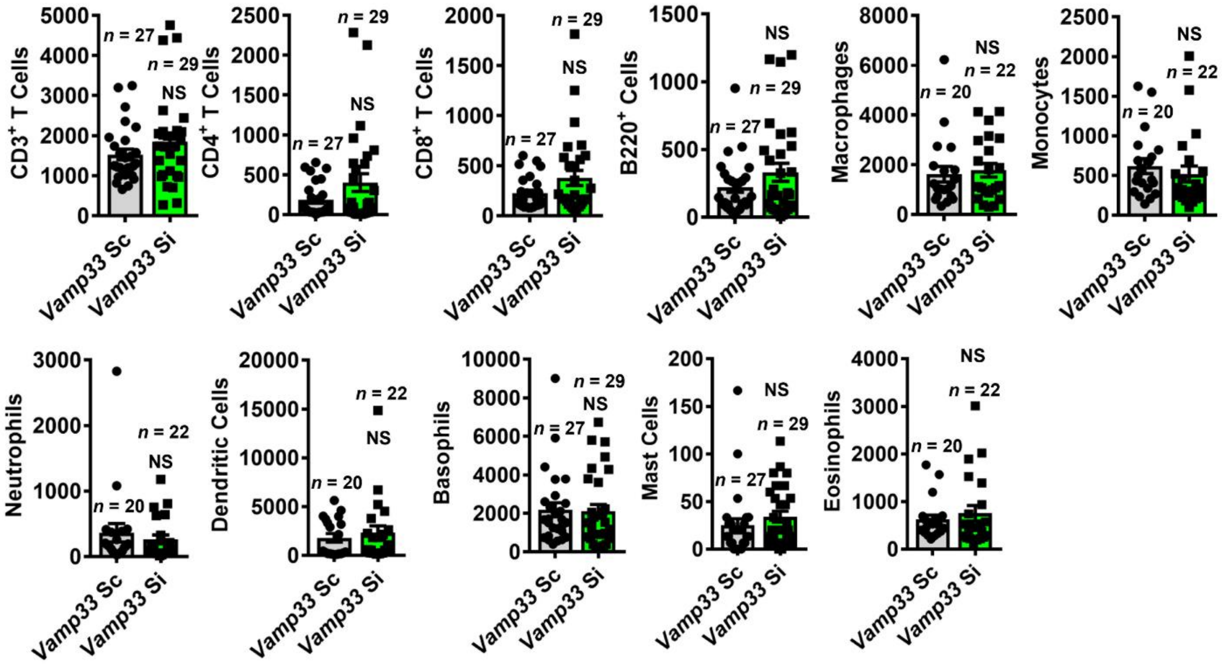


**Supplementary Figure 8.** Immune cell populations at the bite site during *I. scapularis* feeding. Ticks were microinjected with *vamp33* small interfering RNA (*vamp33 si*) (green) or scrambled RNA (*vamp33 sc*) (gray) to reduce the EV release. Ticks were placed on C57BL/6 mice and allowed to feed for three days. Biopsies were taken from the skin and immune cell populations were assessed by flow cytometry. Data are presented as a mean and standard error of the mean (± SEM). Statistical differences in treatments were evaluated by the Student's two-tailed *t* test. (*$p$<0.05; NS = not significant); $n$ = number of samples analyzed. Graphs represents three independent experiments. Source data are provided as a Source Data file. Gating strategy in flow cytometry experiments is shown in Supplementary Fig. **12**.

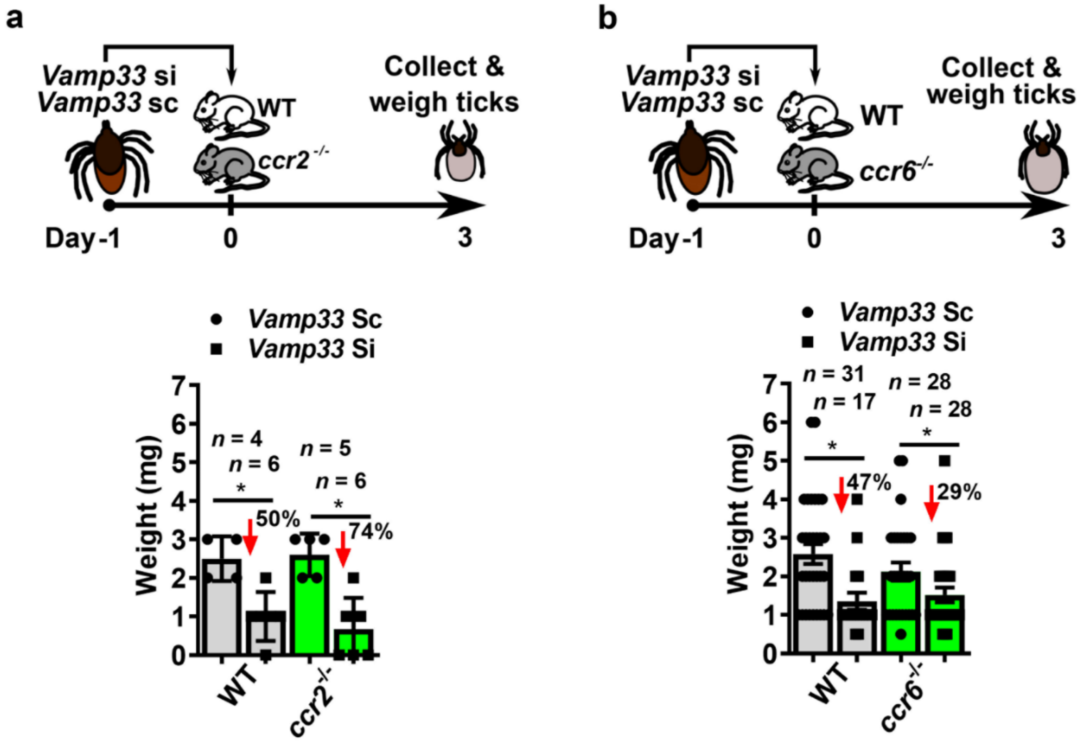


**Supplementary Figure 9.** CCR2- and CCR6-dependent chemokines do not play any role in tick feeding during the release of EVs. **a** and **b** Ticks were microinjected with scrambled RNA (*vamp33 sc*) (circles) or *vamp33* small interfering RNA (*vamp33 si*) (squares) to reduce *I. scapularis* release of EVs during feeding. Ticks were placed on C57BL/6 (WT) mice (gray) and **a** *ccr2*$^{-/-}$ or **b** *ccr6*$^{-/-}$ mice (green) and allowed to feed for three days. Tick weight measurement was then obtained post-infestation. Data is presented as the mean ± SEM. Differences between treatments were evaluated with two-tailed Student's *t* test. (**a** WT $p$=0.005 and *ccr2*$^{-/-}$ $p$=0.001; **b** WT $p$=0.0026 and *ccr6*$^{-/-}$ $p$=0.048 ); $n$ = number of samples analyzed. Source data are provided as a Source Data file.

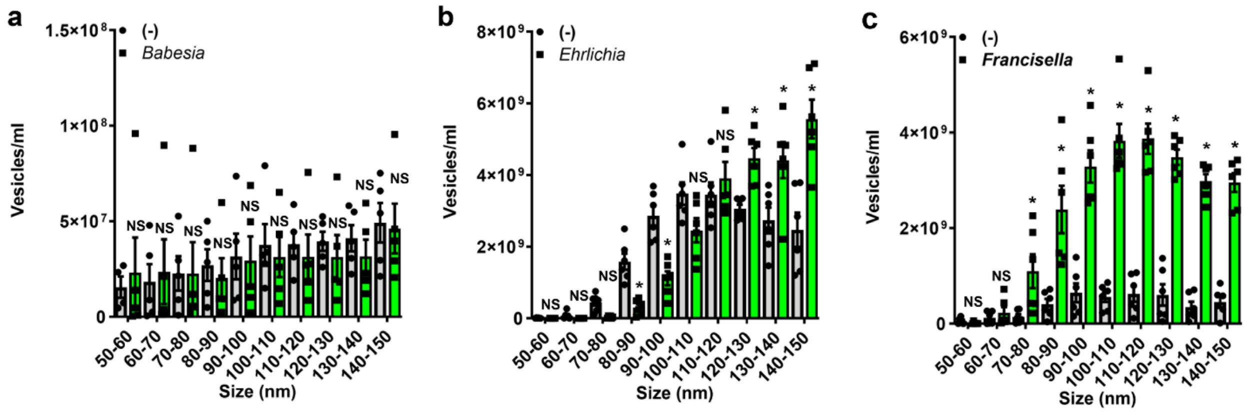


**Supplementary Figure 10.** Tick EVs are repurposed during intracellular bacterial infection. **a** *I. scapularis* ISE6 cells ($2x10^7$) were inoculated with red blood cells infected with *Babesia microti* ($1x10^5$) and EVs were measured 24 hours later. Control ISE6 cells (-) did not receive the parasite inoculum. EVs were then quantified by nanoparticle tracking analysis. No differences were observed between control (gray) and *B. microti*-inoculated (green) cells. The graph is representative of two independent experiments. In **b** and **c**, *A. americanum* AAE2 ($4.7x10^6$) and *D. andersoni* DAE100 tick cells ($6.9x10^6$) were infected with *E. chaffeensis* [multiplicity of infection (MOI) 50] and *F. tularensis* (MOI 10), respectively. EVs release was measured 24 hours later. Control tick cells (-) did not receive the bacterial inoculum. Two-way ANOVA using size and treatment followed by Sidak's multiple comparison statistical tests were used. *$p<0.05$. NS = not significant. The data is presented as the mean ± SEM. Source data are provided as a Source Data file. *n* = technical replicates.

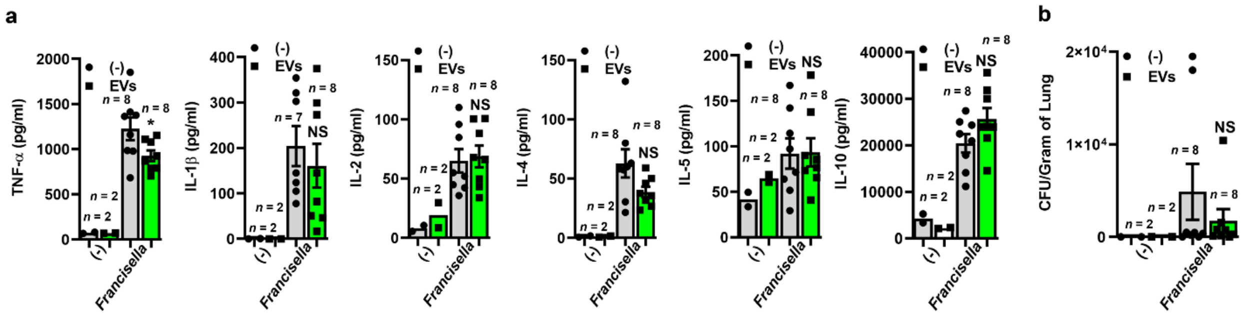


**Supplementary Figure 11** Tick EVs control microbial-induced morbidity and mortality. In **a** cytokine release and **b** *F. tularensis* infection in the mouse lungs were measured by a multiplex cytokine ELISA and colony forming units (CFU) plaque assays at day 5 post-infection, respectively. *F. tularensis* was co-injected in the presence (green) or absence (gray) of *D. andersoni* salivary gland EVs (1x$10^8$). Statistics for the cytokine and infection experiments were done with the Student's two-tailed *t* test in *Francisella* and *Francisella* + EVs samples. Data is presented as a mean and standard error of the mean (± SEM). *$p<0.05$; NS = not significant. All graphs are representative of two independent experiments. Source data are provided as a Source Data file.

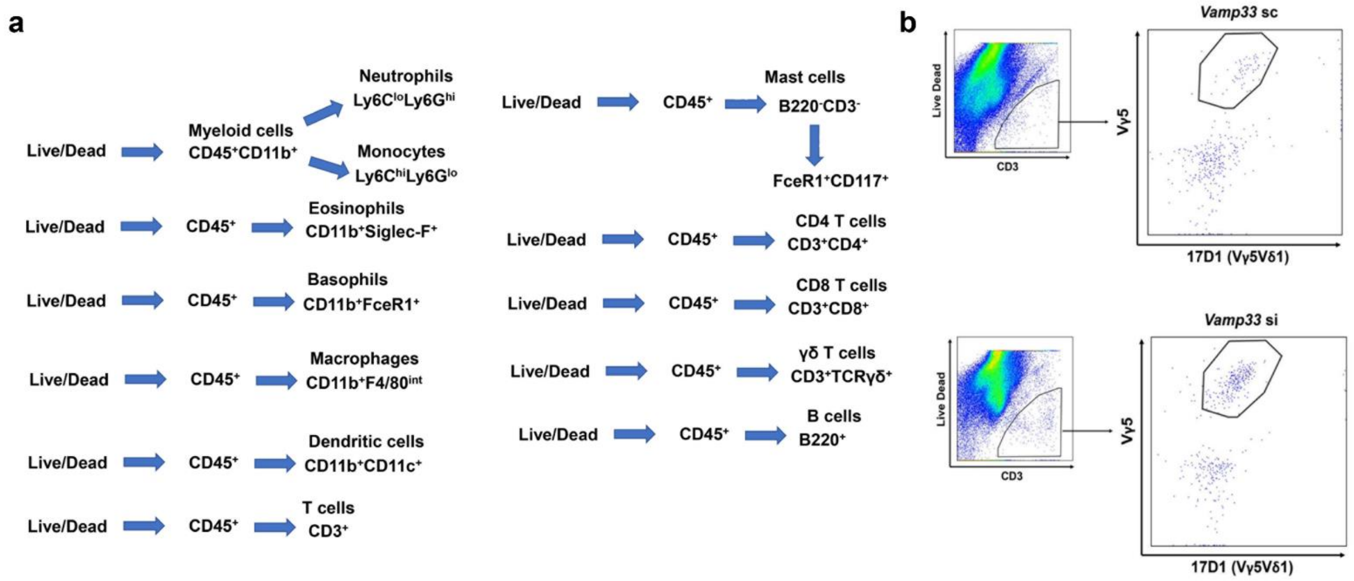


**Supplementary Figure 12.** Gating strategy in flow cytometry experiments. (**a**) Data are shown in Figs. 3**f**, 4**d**, **e** and **g** and Supplementary Fig. **8**. (**b**) Representative flow plots of DETC (Vγ5Vδ1) first gated on live and $CD3^+$ T cells, then stained with the monoclonal antibodies Vγ5 and 17D1(Vγ5Vδ1). Data are shown in Fig. 4**f**. Skin samples were harvested on day 3 from a scrambled RNA (*vamp33 sc*) microinjected tick bite and compared to the *vamp33 si* treatment. Source data are provided as a Source Data file.
