# Supplementary material for: Tick extracellular vesicles enable arthropod feeding and promote distinct outcomes of bacterial infection
Source: Nat Commun. 2021 Jun 17;12:3696. doi: 10.1038/s41467-021-23900-8 (PMC8211691; doi:10.1038/s41467-021-23900-8)
Supplement: Supplementary file 3 — Description of Additional Supplementary Files [file 41467_2021_23900_MOESM3_ESM.pdf]

Description of Additional Supplementary Files

Supplementary Data 1. *In silico* reconstruction of the extracellular vesicle pathway in *Ixodes scapularis* ticks. *I. scapularis, Mus musculus,* and *Drosophila melanogaster* orthologs for proteins involved in the EV biogenesis were identified using the PSI-BLAST tool in NCBI. Human proteins were used as query.

Supplementary Data 2. High-throughput proteomics analysis of cargo proteins present in extracellular vesicles originated from *I. scapularis* salivary glands. Cargo of salivary gland EVs identified by MS/MS mass spectrometry were blasted against the genome of *Homo sapiens* to identify orthologs.

Supplementary Data 3. MS/MS spectra of peptides identified as "1 unique/protein ID" in the proteomics analysis of *Ixodes scapularis.* Peptides and proteins were exported from PEAKS X+ with "–logp>20" scores for peptides and proteins; corresponding to *p*-values<0.01 for both peptides and proteins identification.

Supplementary Data 4. Gene code and protein identification for FunRich analysis. Gene codes from human orthologs to tick proteins identified in salivary gland vesicles were input into FunRich. Data represents the codes of proteins found within the vesiclepedia database.

Supplementary Data 5. Ingenuity pathway analysis of the protein cargo originated from *I. scapularis* extracellular vesicles. Proteins identified with MS/MS were input into the Ingenuity Pathway Analysis software to determine over-represented pathways.

Supplementary Data 6. Primers used for RNAi silencing and qRT-PCR. Primer type, sequence, and location within the target gene are listed.

Supplementary Data 7. Resources and reagents available. Sources, vendors, and dilutions (when applicable) used during these studies.

Supplementary Movie 1. Nanoparticle tracking analysis of nanovesicles purified from *Amblyomma americanum* AAE2 cells.

Supplementary Movie 2. Nanoparticle tracking analysis of nanovesicles purified from *Ixodes scapularis* ISE6 cells.

Supplementary Movie 3. Nanoparticle tracking analysis of nanovesicles purified from *Dermacentor andersoni* DAE100 cells.

Supplementary Movie 4. Nanoparticle tracking analysis of nanovesicles purified from ISE6 cells transfected with the scrambled RNA against the *I. scapularis* gene *vamp33*.

Supplementary Movie 5. Nanoparticle tracking analysis of nanovesicles purified from ISE6 cells transfected with the small interfering RNA against the *I. scapularis* gene *vamp33*.

Supplementary Movie 6. Nanoparticle tracking analysis of nanovesicles purified from ISE6 cells transfected with the scrambled RNA against the *I. scapularis* gene *synaptobrevin 2*.

Supplementary Movie 7. Nanoparticle tracking analysis of nanovesicles purified from ISE6 cells transfected with the small interfering RNA against the *I. scapularis* gene *synaptobrevin 2*.

Supplementary Movie 8. Nanoparticle tracking analysis of nanovesicles purified from *I. scapularis* salivary glands.

Supplementary Movie 9. Live cell imaging of nanovesicles derived from *I. scapularis* ISE6 cells bound to murine bone-marrow derived macrophages.

Supplementary Movie 10. Nanoparticle tracking analysis of nanovesicles purified from *I. scapularis* ISE6 uninfected cells.

Supplementary Movie 11. Nanoparticle tracking analysis of nanovesicles purified from *I. scapularis* ISE6 cells infected with the intracellular bacterium *Anaplasma phagocytophilum*.

Supplementary Movie 12. Nanoparticle tracking analysis of nanovesicles purified from *I. scapularis* ISE6 cells stimulated with the extracellular bacterium *Borrelia burgdorferi*.

Supplementary Movie 13. Nanoparticle tracking analysis of nanovesicles purified from *I. scapularis* uninfected ISE6 cells.

Supplementary Movie 14. Nanoparticle tracking analysis of nanovesicles purified from *I. scapularis* ISE6 cells infected with the parasite *Babesia microti*.

Supplementary Movie 15. Nanoparticle tracking analysis of nanovesicles purified from *D. andersoni* uninfected DAE100 cells.

Supplementary Movie 16. Nanoparticle tracking analysis of nanovesicles purified from *D. andersoni* DAE100 cells infected with *Francisella tularensis*.

Supplementary Movie 17. Nanoparticle tracking analysis of nanovesicles purified from uninfected *Amblyomma americanum* AAE2 cells.

Supplementary Movie 18. Nanoparticle tracking analysis of nanovesicles purified from *Amblyomma americanum* AAE2 cells infected with *Ehrlichia chaffeensis*.

Supplementary Movie 19. Nanoparticle tracking analysis of nanovesicles purified from *Dermacentor andersoni* salivary glands.
