# Supplementary material for: Tick extracellular vesicles enable arthropod feeding and promote distinct outcomes of bacterial infection
Source: Nat Commun. 2021 Jun 17;12:3696. doi: 10.1038/s41467-021-23900-8 (PMC8211691; doi:10.1038/s41467-021-23900-8)
Supplement: Supplementary file 30 — Reporting Summary [file 41467_2021_23900_MOESM30_ESM.pdf]

nature research

Corresponding author(s): Joao HF Pedra

Last updated by author(s): May 6, 2021

Reporting Summary

Nature Research wishes to improve the reproducibility of the work that we publish. This form provides structure for consistency and transparency in reporting. For further information on Nature Research policies, see our Editorial Policies and the Editorial Policy Checklist.

Statistics

For all statistical analyses, confirm that the following items are present in the figure legend, table legend, main text, or Methods section.

n/a	Confirmed	
☐	☒	The exact sample size (*n*) for each experimental group/condition, given as a discrete number and unit of measurement
☐	☒	A statement on whether measurements were taken from distinct samples or whether the same sample was measured repeatedly
☐	☒	The statistical test(s) used AND whether they are one- or two-sided<br>*Only common tests should be described solely by name; describe more complex techniques in the Methods section.*
☐	☒	A description of all covariates tested
☐	☒	A description of any assumptions or corrections, such as tests of normality and adjustment for multiple comparisons
☐	☒	A full description of the statistical parameters including central tendency (e.g. means) or other basic estimates (e.g. regression coefficient) AND variation (e.g. standard deviation) or associated estimates of uncertainty (e.g. confidence intervals)
☐	☒	For null hypothesis testing, the test statistic (e.g. *F*, *t*, *r*) with confidence intervals, effect sizes, degrees of freedom and *P* value noted<br>*Give P values as exact values whenever suitable.*
☒	☐	For Bayesian analysis, information on the choice of priors and Markov chain Monte Carlo settings
☒	☐	For hierarchical and complex designs, identification of the appropriate level for tests and full reporting of outcomes
☒	☐	Estimates of effect sizes (e.g. Cohen's *d*, Pearson's *r*), indicating how they were calculated

Our web collection on statistics for biologists contains articles on many of the points above.

Software and code

Policy information about availability of computer code

Data collection	NTA data was collected as using NTA software versions 2.0 or 3.0 as pdf and excel files. Western blot images were collected using Image Lab Software version 5.0. TEM images were taken using AMT600 software. Cytokine and chemokine data was acquired using MSD discovery workbench. Quantitate PCR was collected in the Applied Biosystems 7500 Fast Real-Time PCR System through the 7500 Software v2.3 or the Bio-Rad CFX96 Real-Time System through the CFX Maestro Software 1.1. Time-lapse imaging was collected in the Olympus VivaView Microscope through the MetaMorph software. Confocal microscopy was done by using the Zeiss LSM 710 microscope and the Zen software. Proteomics data acquisition was performed using the instrument provided Xcalibur™ (version 3.0) software. An independent validation of the MS/MS-based peptides and protein identification was performed with the Scaffold (version Scaffold_4.6.2 and 4.10.0, Proteome Software Inc.) using the compatible ".mzid" files of each sample exported from PEAKS versions 8.5 and X+.
Data analysis	Data was analysed using GraphPad from Prism. Microscopy and TEM images were analysed using ImageJ. Flow data was analyzed with FlowJo or FCS Express 6 Flow Research Edition (FlowJo software v 10.6.1 (Treestar)). Proteomics data was analyzed with Peaks 8.5/X+, ingenuity pathway analysis (IPA), PSI-BLAST, and FunRich. Cytokine and chemokine data was analyzed using MSD discovery workbench– methodical mind version 2013-2019.

For manuscripts utilizing custom algorithms or software that are central to the research but not yet described in published literature, software must be made available to editors and reviewers. We strongly encourage code deposition in a community repository (e.g. GitHub). See the Nature Research guidelines for submitting code & software for further information.

Data

Policy information about availability of data

All manuscripts must include a data availability statement. This statement should provide the following information, where applicable:

- Accession codes, unique identifiers, or web links for publicly available datasets
- A list of figures that have associated raw data
- A description of any restrictions on data availability

The mass spectrometry proteomics data have been deposited to the ProteomeXchange Consortium via the PRIDE 99 partner repository with the dataset identifier PXD018779 under the project name “Label free proteomics profiling of nanovesicle isolated from cultured salivary glands isolated from partially-fed adult female Ixodes scapularis” - Project DOI: 10.6019/PXD018779. All other data are available upon reasonable request.

Field-specific reporting

Please select the one below that is the best fit for your research. If you are not sure, read the appropriate sections before making your selection.

[x] Life sciences [ ] Behavioural & social sciences [ ] Ecological, evolutionary & environmental sciences

For a reference copy of the document with all sections, see nature.com/documents/nr-reporting-summary-flat.pdf

Life sciences study design

All studies must disclose on these points even when the disclosure is negative.

Sample size	Sample size was determined based on statistical analysis. Two or three independent experiments were combined depending on statistical analysis.
Data exclusions	Outliers were detected by a Graphpad Quickcals program (https://www.graphpad.com/quickcalcs/Grubbs1.cfm). Those samples with statistically confirmed as outliers with an alpha of 0.05 were excluded prior to analysis.
Replication	All experiments were performed at least two times unless otherwise specified in the figure legend.
Randomization	Animals and ticks were randomly selected into different experimental groups with equal "n" number prior to the beginning of the experiments. All data collection was randomized and separated by experimental treatments.
Blinding	No blinding occurred during these studies. Experimental design did not require blinding because assessed variables are not confounded by the evaluator. We only focused on measurable variables (weight, number of cells, gene expression, etc).

Reporting for specific materials, systems and methods

We require information from authors about some types of materials, experimental systems and methods used in many studies. Here, indicate whether each material, system or method listed is relevant to your study. If you are not sure if a list item applies to your research, read the appropriate section before selecting a response.

Materials & experimental systems

n/a	Involved in the study	
[ ]	[x]	Antibodies
[ ]	[x]	Eukaryotic cell lines
[x]	[ ]	Palaeontology and archaeology
[ ]	[x]	Animals and other organisms
[ ]	[x]	Human research participants
[x]	[ ]	Clinical data
[x]	[ ]	Dual use research of concern

Methods

n/a	Involved in the study	
[x]	[ ]	ChIP-seq
[ ]	[x]	Flow cytometry
[x]	[ ]	MRI-based neuroimaging

Antibodies

Antibodies used

Antibody Source Identifier Dilution
CD63 (Rabbit anti-human) System Biosciences EXOAB-CD63A-1 1:500
TSG101 (Rabbit anti-human) System Biosciences EXOAB-TSG101-1 1:500
ALIX (Rabbit anti-human) System Biosciences EXOAB-ALIX-1 1:500
Calnexin (Rabbit anti-human) Millipore-Sigma AB2301 1:10,000
PGD Polyclonal Antibody (Rabbit anti-human) Thermo Scientific PA5-27486 1:1,000
NRF2 Polyclonal Antibody (Rabbit anti-human) GeneTex GTX55732 1:1,000

Donkey Anti-Rabbit IgG (H+L), HRP affinity purified Novex A16023 1:4,000 - 1:1,000
Rat monoclonal [H139-52.1] Anti-Mouse kappa light chain (HRP) Abcam ab99632 1:4000
APC anti-mouse F4/80 Invitrogen MF48005 1:100
APC/Cy7 anti-human CD11b clone: ICRF44 Biolegend 301341 1:100
APC anti-human CD14 clone: 61D3 eBioscience 17-0149-41 1:100
InVivoMAb anti-mouse TCR γ/δ clone: UC7-13D5 BioCell BE0070 N/A
InVivoMAb polyclonal Armenian hamster IgG BioCell BE0091 N/A
APC/Vio770 anti-mouse CD11c clone: 418 Miltenyi Biotec 130-107-461 3 ng/ul
VioBlue anti-mouse Ly-6G clone: 1A8 Miltenyi Biotec 130-102-227 3 ng/ul
FITC anti-mouse Ly-6C clone: AL-21 Miltenyi Biotec Discontinued 3 ng/ul
PE anti-mouse Siglec-f clone: ES22-10D8 Miltenyi Biotec 130-102-274 3 ng/ul
APC anti-mouse/human CD11b clone: M1/70.15.11.5 Miltenyi Biotec 130-113-793 3 ng/ul
PE/Vio770 anti-mouse F4/80 clone: REA126 Miltenyi Biotec 130-118-459 3 ng/ul
APC/Vio770 anti-mouse CD45 clone: 30F11 Miltenyi Biotec Discontinued 3 ng/ul
eFluor 450 anti-mouse FceR1 alpha clone: MAR-1 eBioscience/Invitrogen 48589882 2 ng/ul
FITC anti-mouse CD45R (B220) clone: RA3-6B2 Miltenyi Biotec 130-102-810 3 ng/ul
PE anti-mouse/human CD11b clone: M1/70.15.11.5 Miltenyi Biotec 130-113-235 3 ng/ul
APC anti-mouse CD117 clone: 3C11 Miltenyi Biotec 130-102-796 3 ng/ul
PE/Vio770 anti-mouse CD3ε clone: 145-2C11 Miltenyi Biotec 130-102-359 3 ng/ul
APC/Vio770 anti-mouse CD8a clone: 53-6.7 Miltenyi Biotec 130-102-305 3 ng/ul
VioBlue anti-CD45 clone: 30F11 Miltenyi Biotec 130-119-130 3 ng/ul
FITC anti-mouse TCRγ/δ clone: GL3 Miltenyi Biotec 130-104-015 3 ng/ul
PE anti-mouse CD4 clone: GK1.5 Miltenyi Biotec 130-102-619 3 ng/ul
APC anti-mouse CD45R (B220) clone: RA3-6B2 Miltenyi Biotec 130-102-259 3 ng/ul
PE/Vio770 anti-mouse CD3 clone: REA641 Miltenyi Biotec 130-116-530 3 ng/ul
PE anti-Mouse TCR β Chain clone: H57-597 BD 553172 2 ng/ul
PE anti-mouse CD45R (B220) clone: RA3-6B2 Miltenyi Biotec 130-102-292 3 ng/ul
APC Cy7 anti-CD45 clone: REA737 Miltenyi Biotec 130-110-662 3 ng/ul
VioBlue anti-mouse CD4 clone: REA604 Miltenyi Biotec 130-118-568 3 ng/ul
FITC anti-mouse CD8a clone: 53-6.7 Miltenyi Biotec 130-118-329 3 ng/ul
PerCP Cy 5.5 anti-mouse TCRγ/δ clone: REA633 Miltenyi Biotec 130-117-665 3 ng/ul
PECy7 anti-mouse CD3ε clone: REA641 Miltenyi Biotec 130-116-530 3 ng/ul
Zombie Violet Fixable Live Dead stain Biolegend 423113 1:500
Anti-mouse CD16/32 Antibody Biolegend 156603 1:500
APC anti-mouse CD45 Antibody clone: 30-F11 Biolegend 103111 1:100
FITC anti-mouse CD3 Antibody clone:17A2 Biolegend 100203 1:100
BV650 anti-mouse Vγ3 Antibody clone: 536 BD 743241 1:50
PE Mouse Anti-Rat IgM clone:G53-238 BD "553888" 1:100
Monoclonal antibody 17D1 Adrian Hayday, Kings College London and Robert Tigelaar, Yale University 1:50
Pls3 polyclonal antibody Sigma SAB2700266-100UL 1 ng/ul
Dhx16 polyclonal antibody Invitrogen PA530272 1 ng/ul
Cct7 polyclonal antibody Proteintech 15994-1-AP 1 ng/ul
Gfpt1 polyclonal antibody Proteintech 14132-1-AP 1 ng/ul
Beta catenin (CTNNB1) monoclonal antibody (clone 6f9) Invitrogen MA1300 1 ng/ul
Phosphoserine antibody Abcam ab9332-100ug 1 ng/ul
DNPH antibodies come within the following kits:
OxiSelect™ Protein Carbonyl Immunoblot kit Cell Biolabs STA-308-T (1:1000 dilution for antibody)
OxiSelect™ Protein Carbonyl ELISA kit Cell Biolabs STA-310 (1:1000 dilution for antibody)

Validation

FlowCytometry antibodies for DETC experiments were validated in previous publications, such as Mallick-Wood CA, Lewis JM, Richie LI, Owen MJ, Tigelaar RE, Hayday AC. Conservation of T cell receptor conformation in epidermal gammadelta cells with disrupted primary Vgamma gene usage. Science 279, 1729-1733 (1998) and Liu H, Archer NK, Dillen CA, Wang Y, Ashbaugh AG, Ortines RV, Kao T, Lee SK, Cai SS, Miller RJ, Marchitto MC, Zhang E, Riggins DP, Plaut RD, Stibitz S, Geha RS, Miller LS. Staphylococcus aureus Epicutaneous Exposure Drives Skin Inflammation via IL-36-Mediated T Cell Responses. Cell Host Microbe. 2017 Nov 8;22 (5):653-666.e5. doi: 10.1016/j.chom.2017.10.006. PMID: 29120743; PMCID: PMC5774218.

Antibodies against extracellular vesicles were validated using HL-60 vesicles as positive control. Specificity of the antibodies against the proteins was validated using HL-60 vesicles and lysates as positive control. Cross-reactivity to the species was tested with tick cell lysates and extracellular vesicles before using in extracellular vesicles from salivary glands.
Cross-reactivity of polyclonal antibodies was validated in tick ISE6 cells, using human cells (HL-60 cells as positive controls). Cross-reactivity was tested in silico by BLAST and the presence of potential epitopes.

The secondary antibodies were tested in blots without proving using primary antibodies. The membranes were developed and the lack of bands was confirmed visually.

Eukaryotic cell lines

Policy information about cell lines

Cell line source(s)	HL-60 promyelocytic leukemia cell line ATCC<br>DH82 canine macrophage provided by Jere McBride, University of Texas Medical Branch<br>Ixodes scapularis embryonic 6 (ISE6) cells provided by Ulrike Munderloh, University of Minnesota<br>Dermacentor andersoni embryonic 100 (DAE100) cells provided by Ulrike Munderloh, University of Minnesota<br>Amblyomma americanum embryonic 2 (AAE2) cells provided by Ulrike Munderloh, University of Minnesota
Authentication	HL-60 cells were received authenticated from ATCC.<br>ISE6 cells have been authenticated through whole genome analysis in Miller et al, 2018 F1000Res and proteomics in Oliver et al 2015, Exp Appl Acarol. IDE12 has been authenticated in liver et al 2015, Exp Appl Acarol by proteomics. We did not authenticate these cell lines.<br><br>DH82 have been authenticated by Dr. Jere McBride. We did not authenticate this cell line.
Mycoplasma contamination	All cell lines are monthly tested for Mycoplasma contamination. All cell lines tested negative for Mycoplasma.
Commonly misidentified lines (See ICLAC register)	No commonly misidentified cell line was used in this study.

Animals and other organisms

Policy information about studies involving animals; ARRIVE guidelines recommended for reporting animal research

Laboratory animals	Ixodes scapularis adult partially fed female ticks from BEI resources (tick age was not monitored, since it is not an important variable for these studies).<br>Ixodes scapularis nymph ticks from BEI resources (tick age was not monitored, since it is not an important variable for these studies)<br>Ixodes scapularis nymph ticks Utpal Pal, University of Maryland College Park (tick age was not monitored, since it is not an important variable for these studies)<br>Ixodes scapularis nymph ticks Ulrike Munderloh, University of Minnesota (tick age was not monitored, since it is not an important variable for these studies)<br>Dermacentor andersoni adult partially fed female ticks Glen Scoles, ARS USDA (tick age was not monitored, since it is not an important variable for these studies)<br>Dermacentor variabilis nymph ticks BEI Resources-Centers for Disease Control and Prevention (tick age was not monitored, since it is not an important variable for these studies)<br>Mice C57BL6J (WT) University of Maryland Baltimore. Female and male mice were used. Ages ranged from 3 weeks - 6 weeks, depending on the experiments.<br>B6-TCRdelta- B6.129P2-Tcrdtm1Mom/J The Jackson Laboratory. Only male mice. Ages ranged from 3 weeks - 6 weeks.<br>Tcrb KO - B6.129P2-Tcrbtm1Mom/J The Jackson Laboratory. Only male mice. Ages ranged from 3 weeks - 6 weeks.<br>Anaplasma phagocytophilum University of Maryland Baltimore<br>Borrelia burgdoferi Jon Skare, Texas A&M University Health Science Center<br>Francisella tularensis Stephanie Vogel, University of Maryland Baltimore<br>Francisella tularensis Eileen Barry, University of Maryland Baltimore<br>Francisella tularensis subsp. holarctica CDC Live Vaccine Strain, BEI Resources<br>Ehrlichia chaffeensis Jere McBride, University of Texas Medical Branch<br>Babesia microti Ben Mamoun Chouki, Yale University<br>FVB/NJ mice The Jackson Laboratory. Only male mice. Ages ranged from 3 weeks - 6 weeks.<br>FVB/NTac mice Taconic Biosciences. Only male mice. Ages ranged from 3 weeks - 6 weeks.<br>B6.129S4-Ccr2tm1Ifc/J The Jackson Laboratory. Only male mice. Ages ranged from 3 weeks - 6 weeks.<br>B6.129P2-Ccr6tm1Dgen/J The Jackson Laboratory. Only male mice. Ages ranged from 3 weeks - 6 weeks.<br>C3H/HeN mice Charles River. Female and male mice. Ages ranged from 3 weeks - 6 weeks.<br>New Zealand White Rabbits. 6-10 month-old female animals
Wild animals	No wild animals were used in this study.
Field-collected samples	No samples were collected from the field.
Ethics oversight	I. ricinus feeding experiments were performed in accordance with the Animal Protection Law of the Czech Republic (§17, Act No. 246/1992 Sb) and with the approval of the Akademie Věd České Republiky (approval no. 161/2010). All mouse experiments were carried out under the guidelines approved by the Institutional Biosafety (IBC#00002247) and Animal Care and Use committees (IACUC#0216015 and #0119012) at the University of Maryland School of Medicine and (IACUC#108672 and IBC#108665) at the University of Toledo College of Medicine and Life Sciences according to the National Institutes of Health (NIH) guidelines (Office of Laboratory Animal Welfare (OLAW) #A3200-01, A323-01, and A3270-1). Calf experiments were done in accordance with the guidelines approved by the IACUC at the University of Idaho (IACUC# 2017-59).

Note that full information on the approval of the study protocol must also be provided in the manuscript.

Human research participants

Policy information about studies involving human research participants

Population characteristics	Blood collections were performed on healthy volunteers who provided informed consent. Only health was used as a co-variable to select volunteers during the initial screening by the nurse according to the IRB protocol.
Recruitment	Recruitment was randomly anounced throughout the School of Medicine. Non healthy volunteers were excluded. Since volunteers were only used to obtain blood for cell isolation. Any potential bias from volunteers do not affect any of our results.
Ethics oversight	The protocol was approved by the Institutional Review Board (IRB# HP-00040025) of the University of Maryland School of Medicine and comply with the 21 CFR part 50.

Note that full information on the approval of the study protocol must also be provided in the manuscript.

Flow Cytometry

Plots

Confirm that:

- [x] The axis labels state the marker and fluorochrome used (e.g. CD4-FITC).
- [x] The axis scales are clearly visible. Include numbers along axes only for bottom left plot of group (a 'group' is an analysis of identical markers).
- [ ] All plots are contour plots with outliers or pseudocolor plots.
- [x] A numerical value for number of cells or percentage (with statistics) is provided.

Methodology

Sample preparation

EV-Macrophage interactions
Macrophage Differentiation
Murine - Bone marrow derived macrophages (BMDMs) were generated from C57BL/6J mice. In brief, mice were euthanized using CO2 and femurs were dissected. Bone marrow was flushed by injecting differentiating medium consisting of DMEM supplemented with 30% L929 condition medium, 10% FBS (Gemini Bio-products), 1x penicillin/streptomycin (Corning), and 1x Amphotericin B (Gibco) into one end of the femur with a 25 G needle. Cells were seeded onto 90 mm Petri dish plates and incubated at 37 °C 5% CO2. Additional differentiating medium was added to cells on day 3 after seeding. Cells were incubated for 7 days until completely differentiated.

Human - Human macrophages were differentiated from peripheral blood mononuclear cells (PBMCs). Briefly, human blood was obtained from healthy adult volunteers. PBMCs were purified from 10 ml of EDTA-treated human blood using Ficoll-Paque PREMIUM density 1.007 g/ml (GE Healthcare). Contaminating red blood cells were lysed with ACK lysis buffer for 5 minutes at room temperature (RT). Monocytes were enriched by negative selection using the human Pan Monocyte Isolation Kit (Miltenyibiotec) and LS Columns (Miltenyibiotec). Monocytes were resuspended in RPMI supplemented with 10% FBS (Gemini Bio-products), 55 μM 2-Mercaptoethanol (Gibco), 1 mM Sodium Pyruvate (Gibco), 1x MEM non-essential amino acids (Gibco), and 1x penicillin/streptomycin (Corning). Cells were counted using Trypan blue stain (0.4%; Thermo Fisher Scientific) in a TC20™ Automated cell counter (Bio-Rad) and 2x106 monocytes were seeded into 6 well plates (Sigma). Human recombinant Macrophage Colony-Stimulating Factor (M-CSF; Biolegend) was added to each well at 50 ηg/ml final concentration on days 0, 2, and 4 after seeding. Medium was changed on day 4. Cells were incubated at 37 °C 5% CO2 for 6 days to allow differentiation into M0 macrophages.

Skin cell analysis
A 10-mm skin punch biopsy was taken while ticks were still attached. Skin samples from control mice were taken from matching locations. Single cell suspensions were prepared from each skin sample . Briefly, skin samples were cut into small pieces with sterile surgical scissors and placed into 14 ml FALCON® polypropylene round-bottom tubes containing 3 ml digestion buffer consisting of 90% RPMI-1640 (Quality Biological), 10% Liberase™ TL Research Grade (Roche), and 0.1% DNAse I (Millipore-Sigma). Digestions were carried out for 1 hour and 5 min at 37 °C on constant shaking at 70 rpm. Single cell suspensions were obtained by passing the digested tissues through a 40 μm cell strainer (Corning), homogenizing the tissue with a plunger and flushing cells with 20 ml RPMI-1640. Cells were centrifuged at 2000 RPM for 5 min at 4 °C, resuspended in 1 ml FACS buffer (PBS containing 1% BSA). 200 μl of the suspension were placed into a 96 well U-bottom plate and surface-stained with antibody panels.

Instrument	Measurements were taken using a LSRII flow cytometer (BD) or a MACSQuant flow cytometer (Miltenyi Biotec).
Software	Flow data was analyzed with FlowJo or FCS Express 6 Flow Research Edition.
Cell population abundance	Purity of the Macrophages was determine by the expression of F4/80 in the case of mice and the expression of CD11b in humans.<br><br>Skin samples were conformed of a mixed population of cells. The cell abundance was determine by the percentage or total number of cells per 10 mm skin biopsy.

Gating strategy

The DETC population was first gated on CD3+ and live cells, then, Vγ5+ and Vγ5+Vδ1+ through monoclonal antibodies

☒ Tick this box to confirm that a figure exemplifying the gating strategy is provided in the Supplementary Information.
